# Intraoperative Quantification of Severe Mitral Regurgitation: A Comparative Assessment of Two-Dimensional Flow Convergence, Three-Dimensional Volumetric, and Doppler-Based Methods

**DOI:** 10.3390/jcdd13020098

**Published:** 2026-02-18

**Authors:** Hany R. Elgamal, Volodymyr Protsyk, Massimiliano Meineri, Joerg Ender, Waseem Zakhary

**Affiliations:** Department of Anesthesiology, University Hospital Leipzig, 04289 Leipzig, Germany; hany.elgamal@helios-gesundheit.de (H.R.E.); volodymyr.protsyk@helios-gesundheit.de (V.P.); massimiliano.meineri@helios-gesundheit.de (M.M.); joerg.ender@helios-gesundheit.de (J.E.)

**Keywords:** echocardiography, regurgitant volume, mitral regurgitation, PISA, 3D VCA

## Abstract

Accurate quantification of mitral regurgitation (MR) is central to perioperative decision-making, yet the agreement and interchangeability of commonly used echocardiographic methods remain uncertain. This study evaluated quantitative MR parameters individually and within a multiparametric framework using three-dimensional (3D) vena contracta area (VCA) as an intraoperative reference. In this single-center retrospective analysis, intraoperative echocardiographic data from 85 patients undergoing mitral valve intervention between July 2024 and June 2025 were analyzed. Regurgitant volume (RVol) and regurgitant fraction (RF) were quantified using two-dimensional proximal isovelocity surface area (PISA), a 3D volumetric method, and a Doppler-based continuity equation. Agreement was assessed by Bland–Altman analysis, and categorical concordance was assessed by Cohen’s kappa for individual and multiparametric grading strategies. Agreement between individual quantitative methods was limited, with substantial bias and wide limits of agreement for both RVol and RF, resulting in poor-to-fair concordance for MR severity classification. Incorporation of RVol and RF into multiparametric grading strategies improved concordance. Compared with 3D VCA, multiparametric integration incorporating PISA-derived measures showed the best overall performance, with high accuracy and sensitivity and moderate specificity. These findings indicate limited interchangeability of standalone quantitative echocardiographic methods and support reporting the applied technique and using a multiparametric approach anchored to 3D VCA when cardiac magnetic resonance imaging is unavailable.

## 1. Introduction

The classification of chronic mitral regurgitation (MR) severity is inherently multiparametric; however, regurgitant volume (RVol) and regurgitant fraction (RF) quantification is required when fewer than four severity criteria are available, as per the 2017 American Society of echocardiography (ASE) guidelines [1,2]. This raises key questions regarding the optimal RVol and RF quantification method and their inter-modality agreement.

Historically, the two-dimensional (2D) flow convergence method, commonly referred to as the Proximal Isovelocity Surface Area (PISA) method, has been the first-line technique for estimating RVol [3]. Three-dimensional (3D) echocardiography has emerged as a promising modality, particularly in assessing ventricular volumes and valve orifice areas. While Cardiovascular Magnetic Resonance (CMR) remains the gold standard for cardiac imaging, 3D echocardiography shows advantages over 2D measurements [4,5,6,7,8,9]. An alternative Doppler-based approach utilizes the continuity principle to quantify RVol. Nonetheless, all echocardiographic techniques rely on multiple measurements that can vary depending on image quality, patient anatomy, and technical and operator-dependent factors [10].

The 3D vena contracta area (VCA) is an emerging parameter for assessing MR severity. It directly measures the effective regurgitant orifice area (EROA) using 3D color Doppler, avoiding geometric assumptions, especially in elliptical or irregular orifices and with multiple eccentric jets. Multiple validation studies have shown good agreement between 3D VCA and CMR, supporting the use of 3D VCA as an intraoperative reference standard for orifice-based MR quantification when CMR is not available [8,11,12].

In this single-center retrospective analysis, RVol and RF were quantified using the 2D flow-convergence PISA method, the 3D volumetric method, and the Doppler-based continuity equation method, and MR severity was graded solely based on each measurement. The primary endpoints are (1) to assess the agreement of the graduation of MR based on each of the different quantitative methods and their categorical concordance and (2) to evaluate categorical concordance after integrating each method’s measurements into the guideline-recommended multiparametric (MP) approach. The secondary endpoint examines the concordance between the MP-derived MR grades from each method and the intraoperatively measured 3D VCA (comparative reference).

## 2. Materials and Methods

This retrospective study was conducted at the Leipzig Heart Centre, Germany, between July 2024 and June 2025, following approval from the local ethics committee (No. 395/24) on 19 November 2024.

Inclusion and exclusion criteria

Adult patients who underwent elective mitral valve (MV) interventions (i.e., Surgical MV repair or transcatheter MV edge-to-edge repair) and had complete echocardiographic data were included. Patients were excluded if they were under 18 years old, had more than mild aortic regurgitation, or had an atrial septal defect. Eighty-five patients who met all eligibility criteria were included in the final analysis. Patient records were reviewed using the hospital information system (iMedOne^®^, Deutsche Telekom Healthcare and Security Solutions, Bonn, Germany) and entered into a pseudonymized database.

Image acquisition and measurements

Transesophageal echocardiography (TEE) was performed under balanced general anesthesia using the Philips EPIQ CVx system^®^ and an X8-2T echo probe (Philips Healthcare, Andover, MA, USA). Each patient underwent a comprehensive TEE examination, which is part of the standard practice at the Heart Center Leipzig. All image loops were acquired prior to thoracic incision or groin puncture, with blood pressure and heart rate maintained within 20% of pre-induction values. TEEs were performed by different anesthesiologists, all with extensive echocardiographic experience and board certification.

All TEE 3D datasets were acquired and analyzed offline using QLAB software (version 10; Philips Healthcare, Andover, MA, USA), which enabled semiautomated quantification and multiplanar reconstruction (MPR) for precise measurements. RVol and RF were assessed using three different methods: the 3D volumetric method, the 2D PISA method, and a Doppler-based method referred to as the continuity equation method.

**Figure 1:**
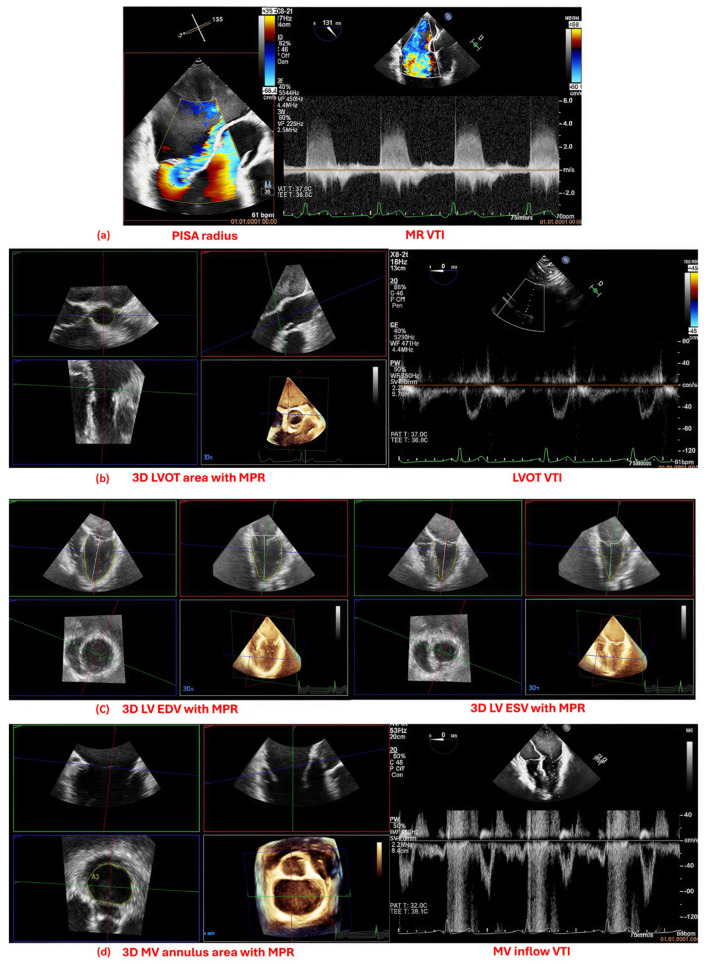
(**a**) PISA method combining hemispheric flow convergence radius with CW Doppler interrogation of the MR jet. (**b**) Forward SV measurement at the LVOT using PW Doppler-derived VTI and 3D LVOT cross-sectional area obtained by MPR. (**c**) 3D LV total SV calculated as the difference between EDV and ESV obtained by MPR. (**d**) Continuity equation method combining PW Doppler assessment of mitral inflow with 3D mitral annular area obtained by MPR. Abbreviations: PISA = proximal isovelocity surface area; CW = continuous-wave; MR = mitral regurgitation; VTI = velocity time integral; SV = stroke volume; LVOT = left ventricular outflow tract; PW = pulsed-wave; 3D = three-dimensional; MPR = multiplanar reconstruction; LV = left ventricular; MV = mitral valve; EDV = end-diastolic volume; ESV = end-systolic volume.

For the 3D volumetric method, RVol was calculated as the difference between 3D total left ventricular (LV) stroke volume (SV) and forward SV. 3D total LV SV was derived from the difference between LV end-diastolic volume (EDV) and LV end-systolic volume (ESV), obtained by analyzing full-volume 3D datasets offline with the 3D Quantification (3DQ) module of QLAB software [10,13]. The end-diastolic and end-systolic frames were identified in orthogonal MPR views as the largest frame following MV closure and the smallest frame preceding MV opening, respectively. To ensure the absence of foreshortening, a maximum LV long-axis length difference of 10% between the four-chamber and two-chamber views was considered acceptable. LV volumes were measured by tracing the endocardial borders at the interface between the LV cavity and the endocardium, including the papillary muscles (Figure 1).

The forward SV was calculated by multiplying the cross-sectional area of the LV outflow track (LVOT) by the LVOT velocity–time integral (VTI) obtained using pulsed-wave (PW) Doppler. The LVOT area was obtained with planimetry at mid-systole from a 3D TEE dataset in the mid-esophageal aortic valve short-axis view, using MPR for orthogonal alignment at the place where the sample volume from PW Doppler was set in the particular patient. The VTI was derived from PW Doppler recordings in the transgastric view (Figure 1).

For the PISA method, the EROA was calculated using the formula EROA = (2πr^2^ × Va)/Vmax, where r is the PISA radius, Va is the aliasing velocity, and Vmax is the peak MR velocity. The RVol was then derived as RVol = EROA × MR VTI. Images were acquired using 2D TEE in mid-esophageal views, applying the conventional hemispherical model [14]. Color Doppler settings were optimized using baseline shift to achieve an aliasing velocity between 30 and 40 cm/s. The PISA radius was measured from the first aliasing contour to the vena contracta. Maximum MR velocity and VTI were measured from continuous-wave Doppler recordings that were aligned with the MR jet direction (Figure 1).

For the continuity equation method, RVol was calculated as the difference between MV SV and forward SV. MV SV was determined by multiplying the VTI of trans-mitral flow—obtained by PW Doppler at the mitral annulus level in mid-esophageal views— multiplied by the MV annular area, which was measured at end diastole from a 3D dataset using MPR to ensure orthogonal alignment at the same level as the Doppler sample volume [1,15] (Figure 1). Forward SV was calculated as described previously in the volumetric method; however, to avoid spurious statistical coupling and correlation, the LVOT cross-sectional area was derived from the LVOT diameter measured by 2D echocardiography in mid-systole in the mid-esophageal aortic valve long-axis view and used instead of the 3D LVOT area, according to the equation: LVOT area = π × (LVOT diameter/2)^2^.

For all Doppler measurements, only data obtained with insonation angles < 20° were accepted to avoid velocity underestimation. In patients with atrial fibrillation, measurements were averaged over three consecutive beats.

The RF for all methods was calculated as the ratio of RVol to total SV. Total SV for each method was calculated consistently as the sum of the RVol derived by the method added to the forward SV.

**Figure 2:**
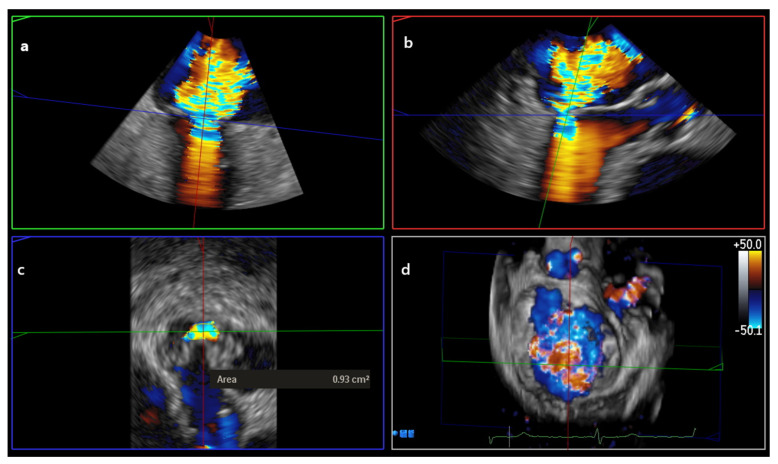
3D VCA measurement derived from MPR of a 3D zoom dataset with color Doppler of the MV. Orthogonal multiplanar reconstruction planes were interactively aligned to the regurgitant jet to ensure true cross-sectional analysis. (**a**) Commissural plane. (**b**) Long-axis plane (**c**) Short-axis plane positioned perpendicular to the jet at the narrowest flow convergence (vena contracta) level, allowing direct planimetric measurement of the VCA. (**d**) En face MV view, Abbreviations: 3D = three-dimensional; VCA = vena contracta area; MPR = multiplanar reconstruction; MV = mitral valve.

VCA was measured using MPR in color 3D Zoom datasets of the MV. The systolic frame displaying the regurgitant jet with the largest VC was selected. The 3D dataset was then rotated to identify two orthogonal long-axis planes and to define the short-axis plane at the level of the vena contracta. In the zoomed short-axis view, the VCA was manually traced along the color–tissue (B-mode) interface [1,11] (Figure 2).

Quantitative RVol/RF Grading:

Severe MR was defined as RVol ≥60 mL for primary MR or ≥45 mL for secondary MR, or RF ≥50%. Patients were then categorized into three groups based solely on the quantitative method used: PISA, Volumetric, and Continuity equation [1,16].

Multiparametric Grading:

According to the 2017 ASE and 2025 ESC/EACTS guidelines [1,16], each patient was evaluated using a multiparametric approach (Table A1). Patients exhibiting four or more predefined echocardiographic parameters were classified as having severe MR without the need for further quantitative assessment. Patients with fewer than four of these parameters were classified according to quantitative parameters, including RVol and RF derived from the PISA, Continuity Equation, or volumetric methods. Patients were then categorized into three groups according to the quantitative method integrated into the MP approach: MP-PISA, MP-Volumetric, and MP-Continuity equation.

3D VCA Grading:

Patients were classified using 3D VCA as a surrogate for EROA. Severity cutoffs were defined as ≥ 0.40 cm^2^ for severe primary MR and ≥ 0.30 cm^2^ for severe secondary MR [1,2,16].

Statistical Analysis:

Sample size: based on the reported ±60 mL limits of agreement between the PISA and volumetric methods in the study by Altes et al. [13] we calculated that 70 paired observations would be required to achieve 95% confidence limits within ±10 mL (α = 0.05). To account for potential attrition, we planned to enroll 85 patients.

The normality of the continuous variables was assessed using the Shapiro–Wilk test. Descriptive statistics are presented as mean ± standard deviation for normally distributed variables or median (interquartile range) for nonnormally distributed data. Categorical variables are expressed as frequencies and percentages.

Comparative Analysis of Echocardiographic Methods: Three complementary statistical approaches were used to compare the three methods—both individually and within a multiparametric framework—overall and in subgroups (primary vs. secondary MR; sinus rhythm vs. atrial fibrillation), each addressing a distinct aspect of performance.

1. Agreement between each pair of methods was assessed using Bland–Altman analysis to examine bias and limits of agreement, providing insight into the closeness of absolute measurements and the magnitude of systematic differences. Differences between paired measurements were plotted against their means to visualize both fixed and proportional bias.

2. Concordance between categorical severity classifications was assessed using Cohen’s kappa for pairwise comparisons among the three quantitative methods, both individually and within the multiparametric framework, as well as in comparison with 3D VCA-based grading. These measures quantify the extent to which different techniques assign patients to the same diagnostic category. Kappa values were interpreted as follows: <0.40 = poor to fair, 0.40–0.75 = moderate to good, > 0.75 = excellent concordance [17,18,19].

3. Diagnostic accuracy was evaluated in a subgroup analysis by assessing the various quantification methods. This included determining the percentage of patients who met ≥4 echocardiographic criteria for severe MR but were classified as non-severe based on quantification alone (using RVol and RF). Additionally, sensitivity and specificity of the multiparametric approach, incorporating PISA, 3D volumetric, and continuity methods, were calculated against 3D VCA grading.

Intra- and interobserver reproducibility was evaluated using the interclass correlation coefficient (ICC) in a randomly selected subset of 10% of patients. Intraobserver reproducibility was assessed by repeating the analysis 15 days after the initial assessment, while interobserver reproducibility was determined by having two independent investigators analyze the same cases.

Statistical analyses were performed with R version 4.3.0 (R Foundation for Statistical Computing, Vienna, Austria) and SPSS version 29.0 (IBM Corp., Armonk, NY, USA).

## 3. Results

A total of eighty-five patients were included. Demographic and preoperative data are described in Table 1.

Comparison of PISA vs. 3D vs. Continuity Equation

**Figure 3:**
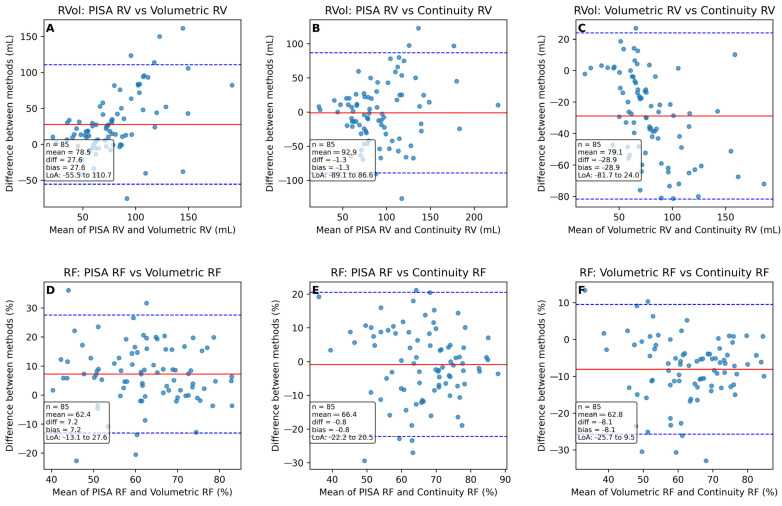
Bland–Altman plots for pairwise comparisons of regurgitant volume (RVol) and regurgitant fraction (RF) measurements. Top row: Bland–Altman plots comparing RV measurements (**A**) PISA vs. Volumetric method (**B**) PISA vs. Continuity methods (**C**) Volumetric vs. continuity method; bottom row: Bland–Altman plots comparing RF measurements (**D**) PISA vs. Volumetric method (**E**) PISA vs. Continuity methods (**F**) Volumetric vs. continuity method. Each plot illustrates the mean difference (bias) and limits of agreement (±1.96 SD) between paired methods. Abbreviations: RVol = regurgitant volume; RF = regurgitant fraction; n = number; LOA (limits of agreement); CI = (confidence interval); PISA = proximal isovelocity surface area.

Bland–Altman analyses demonstrated significant mean biases between methods for RVol and RF, with 3D volumetric quantification yielding lower values compared to PISA and continuity equation approaches (Table A2). The 95% limits of agreement were wide, and proportional bias increased with higher measurement values. Several observations fell outside the limits of agreement (Figure 3). Concordance in severity grading based solely on quantitative measurements was generally poor to fair (Table 2).

MP Comparison:

Applying a multiparametric grading approach improved categorical concordance, with the highest agreement observed between PISA and the volumetric method (κ = 0.62), corresponding to moderate-to-good concordance (Table 2).

MP vs. VCA

Compared with 3D VCA-based grading, the multiparametric approach including PISA performed best, achieving a κ value of 0.54, also indicating moderate-to-good agreement (Table 2).

**Figure 4:**
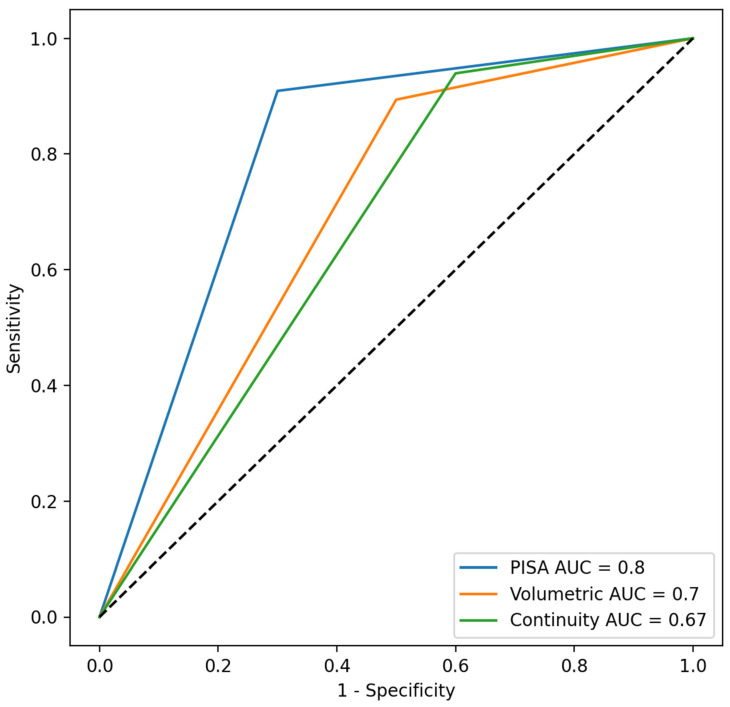
ROC curves comparing the performance of MR severity grading by PISA, volumetric, and the continuity equation methods against the reference standard of 3D VCA. The area under the curve values indicate the diagnostic accuracy of each method for classifying MR severity. Abbreviations: ROC = receiver operating characteristic; MR = mitral regurgitation; PISA = proximal isovelocity surface area; 3D VCA = three-dimensional vena contracta area; RVol = regurgitant volume; RF = regurgitant fraction; n = number; LOA = limits of agreement; CI = confidence interval; PISA = proximal isovelocity surface area.

Classification according to quantification methods within the multiparametric framework was compared to 3D VCA as the reference standard. MP-PISA demonstrated the most balanced diagnostic performance, with the highest accuracy and specificity among the three methods. The volumetric method showed similar accuracy but markedly lower specificity, while the continuity equation method had the highest sensitivity but substantially reduced specificity. These findings are illustrated in the receiver operating characteristic (ROC) curve in Figure 4 and summarized in Table 3.

In subgroup analyses by pathology (primary vs. secondary MR) and rhythm (sinus rhythm vs. atrial fibrillation), the observed patterns were consistent with those in the overall cohort. None of the subgroups demonstrated strong or excellent performance for any of the metrics, and no statistically significant deviations from the overall pattern were observed (Table A3).

All methods showed strong-to-excellent intra-observer reproducibility. Inter-observer agreement was generally good for continuity and volumetric methods, but only moderate for PISA, indicating greater variability between observers for this method (Table A4).

## 4. Discussion

The main findings of our study are as follows: (1) Quantitative MR measures showed poor inter-method agreement, interchangeability, and categorical concordance when used alone for severity grading. (2) Incorporation of these measures within a multiparametric approach improved categorical concordance. (3) When 3D VCA was used as a comparative reference, MP approaches that included PISA provided the most balanced diagnostic performance, demonstrating the highest accuracy and specificity. (4) These results were consistent across clinical subgroups, indicating that variability is intrinsic to the methods rather than patient characteristics.

Current guidelines recommend using multiple qualitative and quantitative parameters to assess MR severity, as relying on a single method has limitations. RVol provides a precise estimation of the volume overload per beat, directly corresponding to the severity of MR, while RF relates RVol to SV, offering a LV volume-specific assessment. However, each method relies on multiple accurate measurements, and even small errors can lead to significant misestimation of MR severity, especially intraoperatively, where altered loading conditions can affect measurement accuracy [1,20].

PISA is notably limited in secondary MR due to its complex jet morphology, such as crescent-shaped orifices and biphasic flow [21], which reduce accuracy and interobserver agreement, as reflected in our findings [22]. In primary MR, selecting the largest PISA radius at a single time point assumes constant systolic flow; however, peak flow typically occurs in mid-systole, often leading to RVol overestimation, and likely contributed to the low specificity compared to 3D VCA observed in our study [23,24,25,26]. Even with angle correction, a prior study reported minimal improvement [27].

The high RVol estimates by the PISA method observed in our study are consistent with previous reports showing that PISA yields higher RVol values compared to both the 3D volumetric method [3,28,29] and CMR, as highlighted in meta-analyses by Skolborg et al. [7]. This overestimation led to PISA-based quantification classifying more patients as having severe MR relative to CMR, with the greatest discordance observed near the severity threshold [4]. In contrast, one study reported lower RVol values with PISA compared to the 3D volumetric method, likely explained by differences in study populations and a relatively small mean RVol below 25 mL [22].

The 3D assessment of LV volumes addresses key limitations of the 2D biplane method, such as geometric assumptions and apical foreshortening. However, despite these advantages, 3D measurements are known to underestimate LV volumes compared to CMR [9]. Additionally, factors such as limited frame rates and challenges in accurately delineating endocardial borders likely contribute to these more conservative volumetric estimations. In our study, this was reflected by the conservative estimation of RVol, a greater number of patients downgraded in the multiparametric grading scheme and the lower sensitivity of the 3D volumetric method in terms of concordance with 3D VCA.

The continuity equation method assumes that SV is equal across the mitral and aortic valves, an assumption that is valid only under specific conditions [13]. Because it requires at least four separate measurements, the method is inherently prone to compounding errors. Accurate determination of mitral SV depends on obtaining the mitral inflow VTI and annular area at precisely the same level. Although we attempted to optimize this by using 3D MPR, precise alignment remains difficult due to the saddle-shaped geometry of the mitral annulus and its pronounced systolic excursion. This motion can shift the Doppler sample volume toward the narrower portion of the orifice, potentially leading to overestimation of mitral SV and RVol accordingly [1,15].

For a more accurate assessment of forward SV, we used 3D MPR to directly planimeter the LVOT area, accounting for the elliptical shape of the outflow tract [13,30]. This approach is supported by prior studies demonstrating reduced bias and improved agreement compared to conventional 2D methods, although SV remains slightly underestimated relative to CMR [6]. However, as previously noted, to avoid statistical coupling and spuriously high agreement between methods, we used an LVOT area derived from the LVOT 2D diameter for the continuity equation-based RVol estimation, rather than re-using the 3D LVOT area used in 3D volumetric RVol estimation.

In our study, the comparison between PISA and volumetric methods revealed limited agreement and interchangeability, consistent with previous reports that similarly demonstrated poor correlation, reflected by low ICCs [3,26] and wide limits of agreement with systematic bias [22,27].

Including RF alongside RVol allows for a more comprehensive assessment of MR severity. RF demonstrated relatively better agreement, supporting its use as a more robust parameter for clinical decision-making.

3D VCA was introduced to overcome the intrinsic limitations of the PISA method [31]. Early applications using transthoracic echocardiography demonstrated a strong correlation between 3D VCA and a multiparametric approach incorporating PISA-derived RVol as observed in our study [32]. Since then, multiple studies have been conducted [11,12]. One study has shown that 3D VCA correlates closely with CMR-derived measures of MR severity, and 3D VCA and its derived RVol were only minimally underestimated compared with CMR, when contrasted with conventional 2D TEE measurements [8].

The relatively higher severity grade concordance observed between PISA-derived RVol and 3D VCA in our study may partly reflect shared methodological characteristics rather than true superiority of either approach. Both methods rely on geometric assessment of the regurgitant orifice during systole and are subject to similar assumptions regarding timing of peak systolic flow, image quality, and operator-dependent analysis. Consequently, this shared dependence may introduce correlated measurement bias when comparing multiparametric strategies that include PISA with those that do not.

3D VCA also carries several important limitations. It is inherently dynamic and governed by the mechanism of MR, exhibiting a biphasic temporal pattern in patients with secondary MR and a predominantly monophasic pattern in those with primary MR [21]. Consequently, measuring a single systolic frame at the time of the largest orifice may lead to overestimation and contribute to interobserver variability. In addition, the MPR process can be technically demanding, particularly in the presence of eccentric or multiple jets, which must be analyzed separately and then summed to obtain the total VCA. This increases both analysis time and the need for operator expertise [3,11,33,34,35]. The lack of guideline-endorsed cut-off values for 3D VCA is also a limitation. Across different studies, various thresholds have been proposed to distinguish severe from non-severe MR using 3D VCA, with values generally >0.40 cm^2^ for primary MR and lower values for secondary MR [32,33,36]. To address this issue in our study, we adopted the established 2D EROA thresholds, given that 3D VCA serves as a surrogate for EROA [1,16].

Although current guidelines recommend a comprehensive multiparametric approach for grading MR severity, our findings highlight important differences in classification behavior when multiparametric grading is compared to 3D VCA as a comparative reference. The observed lower specificity of the multiparametric approach reflects methodological differences between grading strategies rather than definitive misclassification of MR severity. Among the individual techniques, PISA demonstrated the most consistent agreement with 3D VCA. When incorporated into the multiparametric framework, PISA-derived grading showed the highest specificity, though this remained moderate relative to 3D VCA. These findings align with previous studies showing that PISA-derived RVol often correlates poorly with LVEDV, leading to systematic overestimation in patients with normal or small ventricles and resulting in false-positive classifications of severe MR. Such discrepancies contradict physiological expectations, as larger regurgitant volumes are typically associated with LV enlargement, underscoring the need for cautious interpretation of PISA-derived quantitative measurements in clinical practice [27].

Subgroup analyses by MR etiology and rhythm were consistent with the overall results, suggesting that neither the underlying mechanism of MR nor the presence of atrial fibrillation substantially influenced method performance and supporting the notion that differences arise primarily from methodological rather than patient-related factors.

All three methods demonstrated strong-to-excellent intra-observer reproducibility, confirming their reliability when measurements were repeated by a single operator, as well as good inter-observer reproducibility for the volumetric and continuity equation methods. However, inter-observer reproducibility was only moderate for PISA, reflecting its higher sensitivity to individual operator technique.

### Limitations

This study is limited by its retrospective, single-center design and by the absence of a gold-standard reference method such as CMR. Accordingly, 3D VCA should not be interpreted as a gold-standard reference, but rather as an advanced echocardiographic comparator that shares several intrinsic limitations with other quantitative techniques, including operator dependence and temporal variability of the regurgitant orifice. Furthermore, there are currently no guideline-recommended cut-off values specifically validated for 3D VCA, which limits the generalizability of our grading thresholds. Additionally, altered loading conditions under general anesthesia may have influenced MR severity and, consequently, 3D VCA measurements. Beat averaging was constrained by the retrospective nature of the study and the availability of archived intraoperative image loops, with measurements in atrial fibrillation limited to three consecutive beats. Finally, we included only patients with at least moderate MR; therefore, our findings cannot be extrapolated to patients with mild or trivial regurgitation.

## 5. Conclusions

This study suggests that quantitative echocardiographic methods for grading MR severity are not interchangeable. Within a multiparametric framework, PISA-derived RVol showed the most consistent agreement with 3D VCA and the highest overall diagnostic performance. These findings support the continued use of a comprehensive multiparametric assessment, as recommended by current guidelines, in which PISA-based RVol may serve as an important quantitative anchor and 3D VCA as a complementary tool, rather than reliance on any single quantitative metric. Taken together, our results align with and support existing evidence and guideline recommendations. Future studies should validate these observations in larger, multicenter cohorts and incorporate gold-standard reference modalities such as CMR.

## Figures and Tables

**Table 1:** Demographic data.

Sex (Male)	55 (64.7%)
Age (years)	65.5 ± 13.8
EF (%)	59.2 ± 11.3
Pathology (Primary/Secondary)	60/25 (70.6%/29.4%)
Rhythm (SR/AF)	56/29 (65.8%/44.2%)
Intervention (surgery/M-TEER)	63/22 (74.1%/25.9%)

Abbreviations: EF = ejection fraction; SR = sinus rhythm; AF = atrial fibrillation; M-TEER = mitral valve transcatheter edge-to-edge repair.

**Table 2:** Comparison of Categorical Severity Classification Concordance Using Cohen’s Kappa Among Quantitative MR Methods Alone, Within a Multiparametric Framework, and Relative to 3D VCA.

Comparison	Value	95% CI	Interpretation
Quantitative MR Methods Alone			
PISA vs. Volumetric	0.25	0.04 to 0.46	Poor to fair
PISA vs. Continuity	0.14	−0.09 to 0.38	Poor to fair
Volumetric vs. Continuity	0.23	0.02 to 0.43	Poor to fair
Quantitative MR Methods Within a Multiparametric Framework			
MP-PISA vs. MP-Volumetric	0.62	0.36 to 0.81	Moderate to good
MP-PISA vs. MP-Continuity	0.31	0.01 to 0.58	Poor to fair
MP-Volumetric vs. MP-Continuity	0.49	0.22 to 0.74	Moderate to good
The Multiparametric Framework classification Relative to 3D VCA			
3D VCA vs. MP-PISA	0.54	0.23 to 0.78	Moderate to Good
3D VCA vs. MP- Volumetric	0.36	0.06 to 0.63	Poor to fair
3D VCA vs. MP- Continuity	0.37	0.03 to 0.66	Poor to fair

Abbreviations: MR = mitral regurgitation; MP= multiparametric; PISA = proximal isovelocity surface area; 3D VCA = three dimensional vena contracta area.

**Table 3:** Diagnostic Accuracy, Specificity, and Sensitivity of Multiparametric Quantification Methods Compared with 3D VCA Grading.

Comparison	Accuracy	Sensitivity	Specificity
MP-PISA vs. 3D VCA	0.88	0.91	0.70
MP-Volumetric vs. 3D VCA	0.84	0.89	0.50
MP-Continuity vs. 3D VCA	0.87	0.94	0.40

Abbreviations: MP = multiparametric; PISA = proximal isovelocity surface area; 3D VCA = three-dimensional vena contracta area.

## Data Availability

The original contributions presented in this study are included in the article. Further inquiries can be directed to the corresponding author.

## Abbreviations

The following abbreviations are used in this manuscript:

2D	Two-dimensional
3D	Three-dimensional
3DQ	Three-dimensional Quantification
AF	Atrial fibrillation
ASE	American Society of Echocardiography
CMR	Cardiovascular magnetic resonance
EDV	End-diastolic volume
EROA	Effective regurgitant orifice area
ESV	End-systolic volume
ICC	Intraclass correlation coefficient
LV	Left ventricle/left ventricular
LVOT	Left ventricular outflow tract
MP	Multiparametric
MR	Mitral regurgitation
MV	Mitral valve
MPR	Multiplanar reconstruction
PISA	Proximal isovelocity surface area
PW	Pulsed wave
RF	Regurgitant fraction
ROC	Receiver operating characteristic
RVol	Regurgitant volume
SV	Stroke volume
TEE	Transesophageal echocardiography
VCA	Vena contracta area
VTI	Velocity–time integral

## Appendix A

**Table A1:** Echocardiographic Parameters included in the multiparametric approach.

	Parameter ^1^
1	flail leaflet or other severe valvular lesion
2	vena contracta width ≥0.7 cm (Biplane ≥0.8 cm)
3	PISA radius ≥1.0 cm at a Nyquist limit of 30–40 cm/s
4	a central jet occupying >50% of the left atrial area
5	systolic pulmonary vein flow reversal
6	a dilated LV with preserved systolic function

^1^ Severe mitral MR was considered present when at least four specific guideline-defined signs were observed. Abbreviations: PISA = proximal isovelocity surface area; LV = left ventricle.

**Table A2:** Estimated RVol and RF by Quantification Methods.

Method	RVol	RF
PISA	89 ± 40 mL	65 ± 11%
Continuity	97 ± 45 mL	67 ± 12%
Volumetric	65 ± 27 mL	59 ± 12%.

Abbreviations: RVol = regurgitant volume; RF = regurgitant fraction; PISA = proximal isovelocity surface area.

**Table A3:** Subgroup Comparative Analysis:.

Group	Comparison	RVol Grade Kappa	RF Grade Kappa	RVol (mL) BA Bias	RF (%) BA Bias
Primary MR	PISA vs. Volumetric	0.31 (0.05 to 0.54)	0.1 (−0.12 to 0.36)	27.16 (−52.91 to 107.23)	7.31 (−12.36 to 26.98)
Primary MR	PISA vs. Continuity	0.05 (−0.18 to 0.29)	0.16 (−0.1 to 0.55)	−2.37 (−90.35 to 85.6)	−1.48 (−23.26 to 20.3)
Primary MR	Volumetric vs. Continuity	0.21 (−0.04 to 0.46)	0.26 (−0.03 to 0.53)	−29.53 (−81.45 to 22.38)	−8.79 (−26.21 to 8.63)
Secondary MR	PISA vs. Volumetric	−0.13 (−0.26 to 0.0)	0.35 (−0.09 to 0.76)	28.6 (−62.97 to 120.18)	7.1 (−15.15 to 29.35)
Secondary MR	PISA vs. Continuity	0.46 (−0.09 to 1.0)	0.35 (−0.07 to 0.78)	1.38 (−87.78 to 90.53)	0.68 (−19.76 to 21.11)
Secondary MR	Volumetric vs. Continuity	0.19 (−0.14 to 0.65)	0.29 (−0.06 to 0.63)	−27.23 (−83.27 to 28.82)	−6.42 (−24.4 to 11.55)
SR Subgroup	PISA vs. Volumetric	0.29 (0.04 to 0.53)	0.22 (−0.09 to 0.51)	25.56 (−57.54 to 108.67)	6.84 (−14.09 to 27.77)
SR Subgroup	PISA vs. Continuity	0.17 (−0.12 to 0.44)	0.12 (−0.11 to 0.5)	−4.5 (−94.06 to 85.06)	−2.11 (−23.85 to 19.63)
SR Subgroup	Volumetric vs. Continuity	0.22 (−0.05 to 0.48)	0.3 (−0.03 to 0.62)	−30.06 (−82.66 to 22.53)	−8.95 (−26.64 to 8.74)
AF Subgroup	PISA vs. Volumetric	0.04 (−0.25 to 0.44)	0.11 (−0.13 to 0.45)	31.49 (−52.45 to 115.43)	8.04 (−11.34 to 27.41)
AF Subgroup	PISA vs. Continuity	−0.1 (−0.2 to 0.0)	0.46 (0.0 to 1.0)	4.97 (−79.68 to 89.62)	1.6 (−18.5 to 21.7)
AF Subgroup	Volumetric vs. Continuity	0.16 (−0.13 to 0.62)	0.21 (−0.06 to 0.52)	−26.52 (−80.53 to 27.49)	−6.44 (−23.72 to 10.85)

Abbreviations: RVol = regurgitant volume; RF = regurgitant fraction; PISA = proximal isovelocity surface area; SR = sinus rhythm; AF = atrial fibrillation; BA = Bland–Altmann.

**Table A4:** Intra-Observer and Inter-observer Comparisons.

Method	Intra-Observer ICC (95% CI)	Inter-Observer ICC (95% CI)
PISA	0.98 (0.91–1.00)	0.65 (–0.03–0.92)
Continuity	0.98 (0.90–1.00)	0.76 (0.17–0.95)
Volumetric	0.86 (0.50–0.97)	0.79 (–0.00–0.96)

Abbreviations: ICC = intraclass correlation coefficient; PISA = proximal isovelocity surface area.
